# Potential changes in the distribution of *Carnegiea gigantea* under future scenarios

**DOI:** 10.7717/peerj.5623

**Published:** 2018-09-19

**Authors:** Fabio Albuquerque, Blas Benito, Miguel Ángel Macias Rodriguez, Caitlin Gray

**Affiliations:** 1 Science and Mathematics Faculty, Arizona State University, Mesa, AZ, United States of America; 2 Ecological and Environmental Change Research Group, Department of Biological Sciences, University of Bergen, Bergen, Norway; 3 Centro Universitario de Ciencias Biológicas y Agropecuarias, Universidad de Guadalajara, Guadalajara, Mexico

**Keywords:** Biogeography, Species distribution models, Spatial analysis, Climate change, Species distribution, Cactaceae, Saguaro, Sonoran desert

## Abstract

Over the last decades several studies have identified that the directional changes in climate induced by anthropogenic emissions of greenhouse gases are affecting the ecology of desert ecosystems. In the Southwest United States, the impacts of climate change to plant abundance and distribution have already been reported, including in the Sonoran Desert ecosystem, home of the iconic Saguaro (*Carnegiea gigantea*). Hence, there is an urgent need to assess the potential impacts of climate change on the saguaro. The goals of this study are to provide a map of actual habitat suitability (1), describe the relationships between abiotic predictors and the saguaro distribution at regional extents (2), and describe the potential effect of climate change on the spatial distribution of the saguaro (3). Species Distribution Modeling (SDM) was used to investigate the relationships between abiotic variables and the Saguaro distribution. SDMs were calibrated using presence records, 2,000 randomly-generated pseudo absences, and ten abiotic variables. Of these, annual precipitation and max temperature of the warmest month was found to have the greatest relative influence on saguaro distribution. SDMs indicated that 6.9% and 8.1% of the current suitable habitat is predicted to be lost by 2050 and 2070, respectively. Therefore, predicted changes in climate may result in a substantial contraction of the suitable habitat for saguaro over the next century. By identifying the drivers of saguaro distribution and assessing potential changes in habitat suitability due to climate change, this study will help practitioners to design more comprehensive strategies to conserve the saguaro in the face of climate change.

## Introduction

Predicting climate change effects on biodiversity is one the most important challenges that researchers face (Parmesan, 2006). Over the last decades several studies have identified the changes induced by anthropogenic emissions of greenhouse gases is affecting the ecology of desert ecosystems (Parmesan & Yohe, 2003; Kimball et al., 2010). Climate change researchers predict that changes to desert ecosystems will alter the nutrient cycles, fire regimes, genetic diversity of populations with implications to evolutionary changes, and cause species range shifts (Loik et al., 2004; Huxman et al., 2004; Bickford et al., 2010; SDNIMP, 2010). In the Southwest United States, the impacts of climate change on plant abundance and distributions have already been reported, including in the Sonoran Desert (Munson et al., 2012).

The saguaro (*Carnegiea gigantea*) represents one of the most noticeable patterns of plant distribution in the Sonora Desert (Hutto, McAuliffe & Hogan, 1986). The saguaro is a large columnar cactus that grows to a height of 12 m or more. The main stem, which can range up to 75 cm in diameter, has 12 to 25 vertical ribs (Turner, Bowers & Burgess, 1995; Anderson, 2006). This species inhabits rocky and outwash slopes and grows on sandy flats on or near alluvium (Turner, Bowers & Burgess, 1995). The saguaro is very important to the people of the Tohono O’odham Nation, because they rely on this species for food (Beckwith, 2015). The spatial distribution of the saguaro extends through the Sonoran Desert in Arizona, California, and Mexico, but most of its population occurs in Sonora, Mexico (USFR, 2016). Saguaro disease (e.g., bacterial necrosis), air pollution, cattle grazing, wood-cutting, land use changes, urbanization, freezing and drought are significant threats to the saguaro (NPS, 2010; Burquez-Montijo et al., 2013).

However, despite its importance as one of the signature species of the Sonoran Desert, the giant saguaro has been largely ignored by biogeographers. There is limited evidence that the spatial distribution of the saguaro is driven mainly by the climate in the northernmost part of its range (Hutto, McAuliffe & Hogan, 1986; Turner, Bowers & Burgess, 1995; Arundel, 2005). However, the limiting factors affecting the growth of the saguaro in the eastern Sonora State of Mexico have not been identified yet (Turner, Bowers & Burgess, 1995). Hence, there is an urgent need to review our current understanding of the effects of climate on the saguaro distribution.

Species Distribution Modeling (SDM) are correlative models built from the relationships between environmental variables and incomplete presence records (Guisan & Zimmermann, 2000) that have been used to provide understanding on detailed ecological relationships between abiotic predictors and species distributions, and to predict species’ distributions across space and time (e.g., Guisan & Zimmermann, 2000; Araújo & Rahbek, 2006; Elith et al., 2006). The outcome of an SDM is a habitat suitability map, useful for assessing species invasion and proliferation, designing ecogeographic regions, modeling species richness and composition, and supporting conservation planning and spatial prioritization, among others (Ferrier et al., 2002; Franklin, 2010; Benito, Cayuela & Albuquerque, 2013;  Guisan et al., 2013).

When forecasting the effect of climate change on species ‘geographical ranges, it is important to consider multiple climate change scenarios (Sala et al., 2000; Parmesan, 2006; Araújo & Rahbek, 2006; Beaumont et al., 2007; Beaumont, Hughes & Pitman, 2008; Bellard et al., 2012) based on four Representative Concentration Pathways (RCPs, IPCC, 2013). RCPs describe scenarios based on assumptions on socio-economic, and greenhouse and air pollutant emissions to provide trajectories for major agents of climate change (Van Vuuren et al., 2011). In this paper we apply SDMs to investigate how habitat suitability for the saguaro can potentially respond to a range of climate change scenarios. Our results provide guidance on the potential impacts of climate change on the saguaro’s geographical range, while increasing our understanding on the impacts of climate change in the ecology of the Sonora Desert.

## Materials & Methods

**Figure 1:**
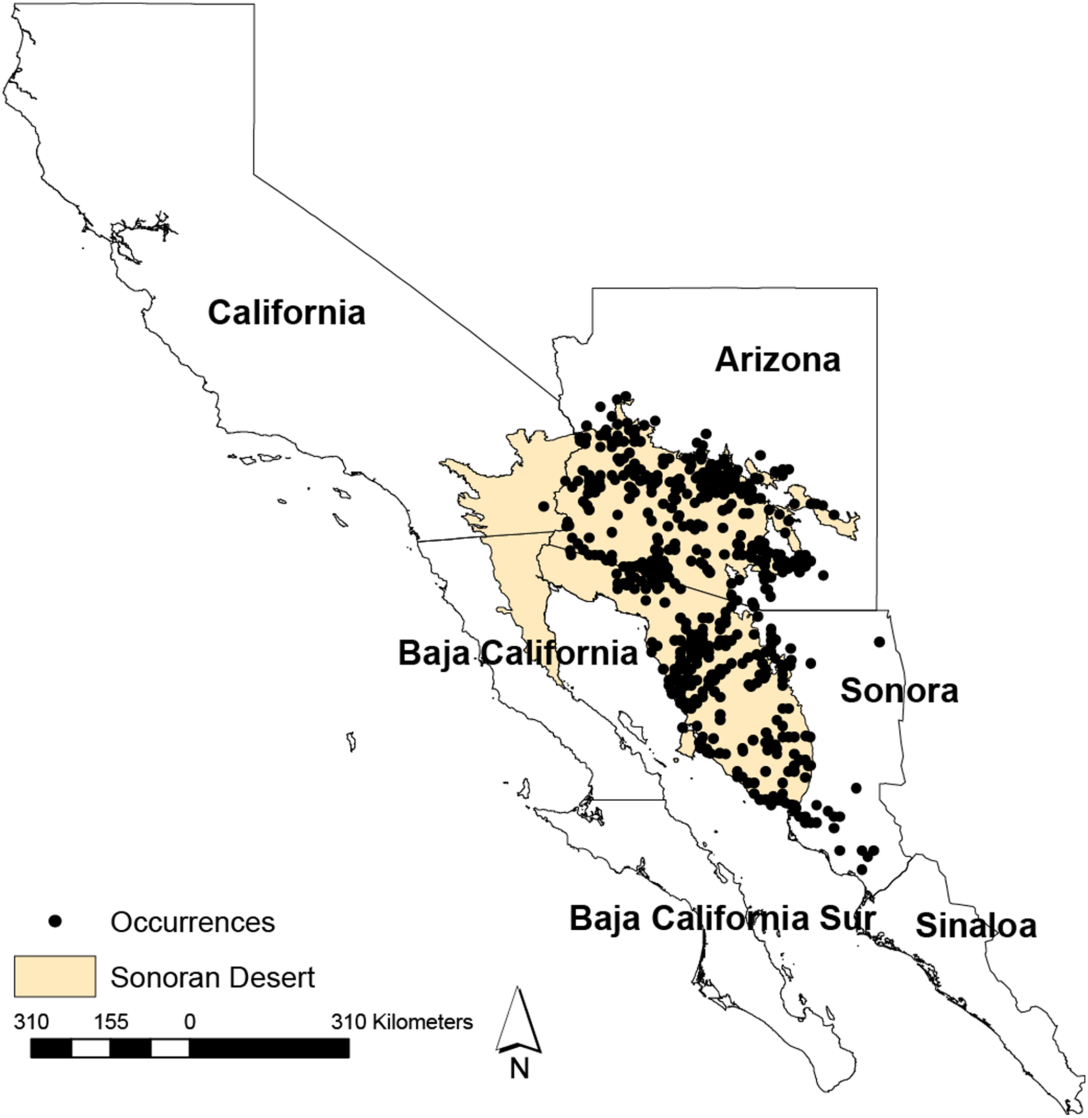
Geographical distribution of the Sonoran Desert and occurrences. Geographical distribution of the Sonoran Desert and occurrences points used in this study. The limits of the Sonoran Desert were extracted from The Nature Conservancy’s core global spatial data (TNC, 2018).

The Study area comprised the Sonora Desert (Fig. 1), which extends from the Southwestern United States into Northern Mexico, including the states of Arizona, California, Baja California, and Sonora. It is rich in both habitat and biodiversity, and encompasses biotic community representing all of the World’s biomes, such as tundra, forest, grassland, chaparral, desert and riparian communities (Arizona-Sonora Desert Museum, 2018). The Sonoran Desert lifeforms include more than 350 birds, 100 reptiles, and more than 2,000 plant species, including the iconic columnar cacti species saguaro (NPS, 2017).

### Data preparation

We obtained 824 records on the saguaro distribution from the GBIF (Global Biodiversity Information Facility; URL: http://www.gbif.org), SEINet Portal Network (http://swbiodiversity.org/seinet/index.php) and the TROPICOS database (Missouri Botanical Garden; URL: http://www.tropicos.com). To prepare a reliable presence dataset, we cleaned the data by (1) removing records with wrong latitudes and longitudes (e.g., records located in the Pacific Ocean); (2) deleting duplicated records; (3) reducing spatial aggregation by imposing a minimum distance among nearby presence records (Benito, Cayuela & Albuquerque, 2013). We generated a set of 2,000 random points not overlapping the presence data to be used as pseudo-absences to fit SDMs.

Because data is often collected at easily accessed areas and bias in the selection of sampling sites can affect model quality (Phillips et al., 2009), we used four target distances (one km, four km, seven km and 10 km) to estimate the optimal minimum distance between consecutive presence and background records. To do so, we first created a regular grid of cells for record sampling. The cell size values are the same as defined by the target distances. We randomly selected one record per grid cell to take a sample of points within the grid cells. We used a stratified random split to split presence and pseudo-absence records into a training dataset (30%) and a testing dataset (70% points).

We included two classes of variables in the models: (1) topographic variables derived from WorldGrids (2018), such as elevation, slope, topographic wetness index, topographic openness index, and potential incoming radiation (mean and range). (2) Climate variables for the present time and future climatic projections from WorldClim (http://worldclim.org/; Hijmans et al., 2005), including annual and seasonal means, extremes, and ranges of temperature and precipitation. A list of all variables used is available at Table S1.

We used future climatic projections produced from two global climate models (GCMs), CCSM4 and HadGEM2-ES. The Community Climate System Model (CCSM4) is a coupled global climate model, simulating Earth’s atmosphere, ice, land, and ocean from the past into the future (Gent et al., 2011). The Hadley Global Environment Model (HadGEM2-ES) is an earth systems model incorporating terrestrial, oceanic, and atmospheric conditions (Jones et al., 2011). Both models have been used extensively in addressing the effects of climate change on species distributions (e.g. Buisson et al., 2010; Naujokaitis-Lewis et al., 2013). The data targets two-time periods: 2050 (average for 2041–2060) and 2070 (average for 2061–2080) at approximately 1 × 1 km resolution and has been generated following each one of the four Representative Concentration Pathways (RCPs) described in the Intergovernmental Panel on Climate Change’s Fifth Assessment Report (IPCC, 2013; Bosso et al., 2016). Each RCP (2.6, 4.5, 6.0 and 8.5) assumes a set of different socioeconomic, technological, and political scenarios, representing optimistic to pessimistic greenhouse gas concentration trajectories.

### Species distribution modeling

The calibration of SDMs requires the following steps: obtaining relevant presence records; selecting relevant predictors; selecting the appropriated numerical model; fitting and evaluating the model from training and test data; and mapping predictions to the geographical space (Elith & Leathwick, 2009).

#### Variable selection

Following Benito, Cayuela & Albuquerque (2013), we computed the correlation matrix among predictors, and used a hierarchical cluster analysis (*hclust* R function) to group predictors according their mutual correlation by setting the maximum correlation at 0.5 Pearson’s index. We identified nine strongly-correlated groups: one related with potential radiation, and eight groups associated with measures of precipitation, temperature, and elevation (Fig. S1). We generated Biserial correlation models (Kraemer, 2006), a special case of Pearson correlation in which one variable is quantitative and the other variable is binomial, to investigate relationships between environmental predictors and the saguaro distribution. For each group identified by *hclust*, we selected the predictor that best correlated with the saguaro distribution. Finally, we used variance inflation factor analysis (VIF) to minimize collinearity among predictors. We considered values of VIF above five as an evidence of collinearity (Heiberger & Holland, 2004). The selected variables were: annual mean temperature, max temperature of warmest month, mean temperature of wettest quarter, annual precipitation, precipitation seasonality, topographic wetness index, topographic openness index, and potential income radiation.

#### Model fitting

We used the training data and the selected environmental variables to fit boosted regression trees models (BRT; Elith, Leathwick & Hastie, 2008), an ensemble algorithm that combines the strengths of two models: decision trees and boosting. The former is known by its ability to (1) handle several types of response variables (e.g., numeric, categoric, multivariate), (2) handle complex interactions, and (3) deal with missing values with minimal loss of information (De’ath, 2007). Boosting is an optimization technique for minimizing the loss function (in this case deviance). The general idea is to generate a sequence of trees, and for each successive step, a tree is built using the residuals of the previous iterations as input (De’ath, 2007; Elith, Leathwick & Hastie, 2008), until residuals stop decreasing. The resulting BRT model is the combination of all the fitted trees, and the prediction is computed from the sum of the output of the individual trees (Elith, Leathwick & Hastie, 2008).

BRT models were calibrated with the function *gbm.step* of the R package *dismo* (Hijmans et al., 2017; R Core Team, 2017). BRT requires the specification of five main parameters: bag fraction (*bf*), learning rate (*lr*), tree complexity (*tr*), step size (*ss*), and number of trees (*nt*). Bag fraction is the percentage of the data randomly selected to build the next tree. Learning rate is used to set the weight applied to individual trees. Smaller *lr* values will increase the number of trees required. Tree complexity represents the number of nodes in a tree. Step size controls the number of trees to add at each running of the boosting algorithm (Derville et al., 2016). To estimate the optimal *nt*, we used different combinations of *bf* (0.1, 0.5, 0.75), *lr* (0.01, 0.005, 0.001), *tc* (1, 2, 3, 4), and *ss* (25, 50), to produce a set of 360 BRT models (Derville et al., 2016).

#### Model evaluation

For each model, we used 10 k-fold cross-validation procedure to split the training data into ten random subsets to estimate the area under the receiver operating characteristic curve value, namely AUC (Area under the curve Fielding & Bell, 1997). We also considered the deviance explained by the model as reported by the *gbm.step* function output. We compared model performance using target-distance and BRT parameters, and we selected the model with highest AUC and deviance explained to determine the optimal *bg*, *lr*, *tc* and *ss* parameters.

We analyzed the relative influence of each variable provided by the *gbm.step* function for the best BRT model, and used the function *gbm.plot* to produce partial dependence plots (Hastie, Tibshirani & Friedman, 2001) showing the relationship between predictor variables and the distribution of the saguaro.

#### Model prediction

The best BRT model was used to forecast habitat suitability in the present time, and over every combination of time period, GCM, and RCP to produce eight future presence range maps. We used the maximization of the sum of sensitivity and specificity statistics to transform habitat suitability as estimated by the best model, into a binary prediction (Liu et al., 2005; Lawson et al., 2014). We followed Hatten et al. (2016) to identify potential range expansion, contraction, or consistency under the four RCPs. For each RCP, we summed the binary GCM maps, resulting a value of 0 where both SDMs predicted absence and a value of two where they predicted presence. We computed potential changes in saguaro habitat suitability between the present and future by subtracting the composite range maps for each period to the present habitat suitability map. We also computed the maps of differences in temperature and rainfall between today and 2070, and calculated the match between the expansion and contraction areas with the maps of differences in temperature and rainfall.

## Results

From the 360 models produced with different combinations of target distance and parameters, models with a target distance of 1km had the best performance. Among them, three models had the same *tc* (4), *bf* (0. 5), and *AUC (0.87)*. We selected the model with the smallest learning rate (*lr* = 0.005).

**Figure 2:**
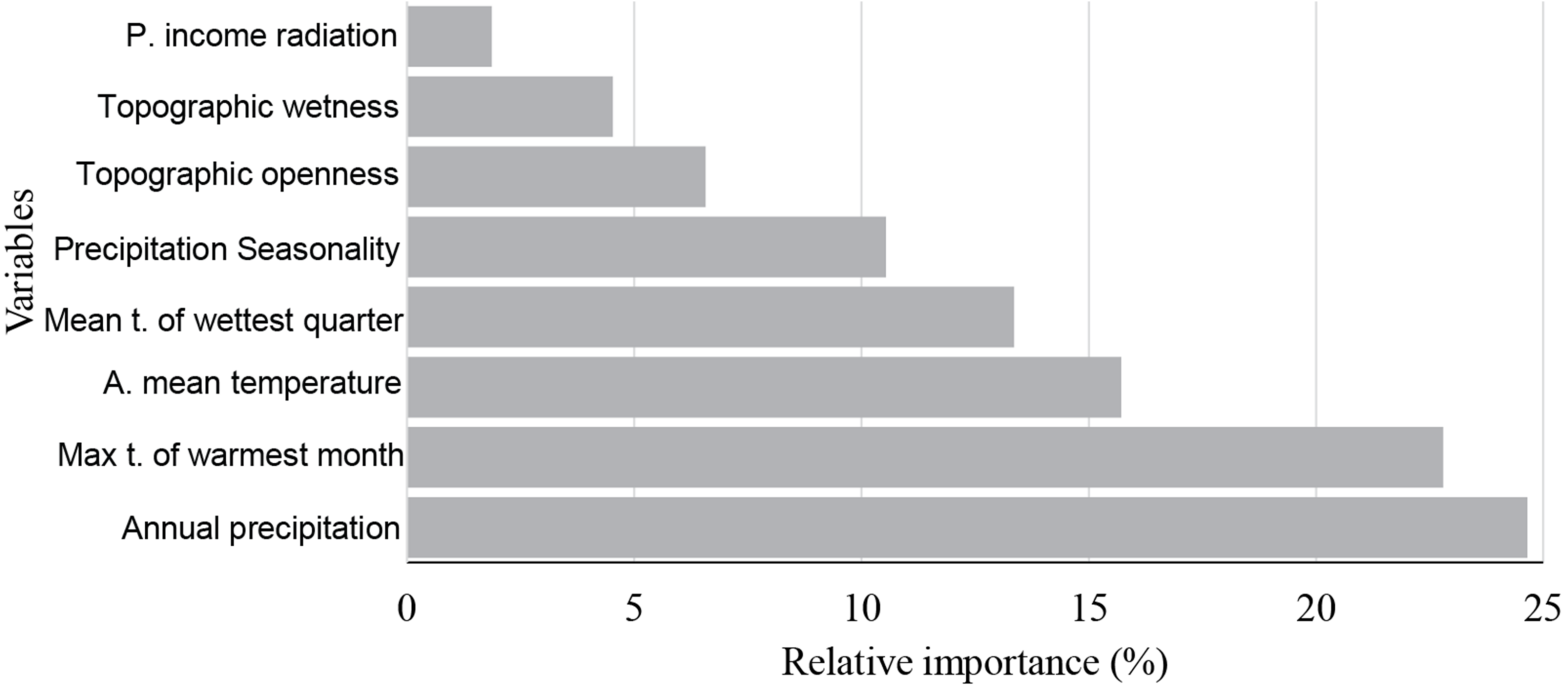
Variable importance measures. Variable importance measures as produced by boosted tree regressions. Variables are annual precipitation, max temperature of warmest month (Max t. of the warmest month), annual mean temperature (A. mean temperature), mean temperature of wettest quarter (Mean t. of the wettest quarter), precipitation Seasonality, topographic openness index, topographic wetness index and potential income radiation (mean, p. income radiation).

According to this model, the annual precipitation and max temperature of warmest month were found to have the greatest relative influence on saguaro’s habitat suitability, 24.7%, and 22.8%, respectively (Fig. 2). Also, the mean annual temperature showed a significant contribution (15.7%).

**Figure 3:**
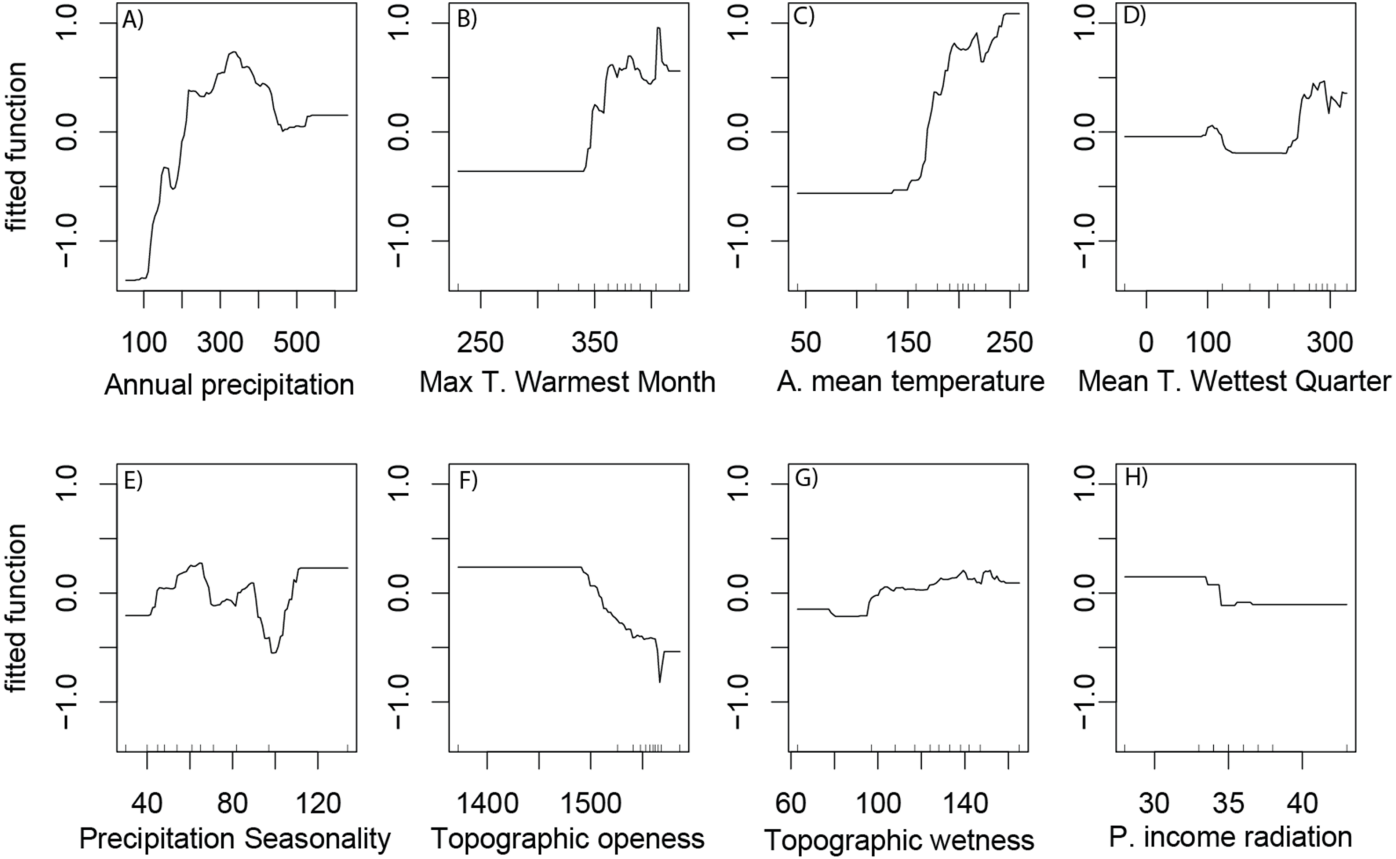
Partial dependence plots. Partial dependence plots representing the marginal effect of environmental variables and human footprint on habitat suitability of Saguaro across the Sonoran Desert. Variables are (A) annual precipitation, (B) max temperature of warmest month (Max t. of the warmest month), (C) annual mean temperature (A. mean temperature), (D) mean temperature of wettest quarter (Mean t. of the wettest quarter), (E) precipitation Seasonality, (F) topographic openness index, (G) topographic wetness index and (H) potential income radiation (mean, p. income radiation). *Y* values are in logit scale and represent the marginal effects of explanation variables on saguaro suitability.

According to the partial dependence plots obtained from the best BRT model, the relationship between saguaro’s habitat suitability and the environmental predictors are non-linear. The partial contribution of the individual predictors to the model fit (Fig. 3) indicate a preference of the species for warmer areas with high precipitation in the summer and open landscapes. For max temperature of warmest month, the logit of the probability of presence displayed a constant response to about 35 °C and then showed a steep increase (Fig. 3).

**Figure 4:**
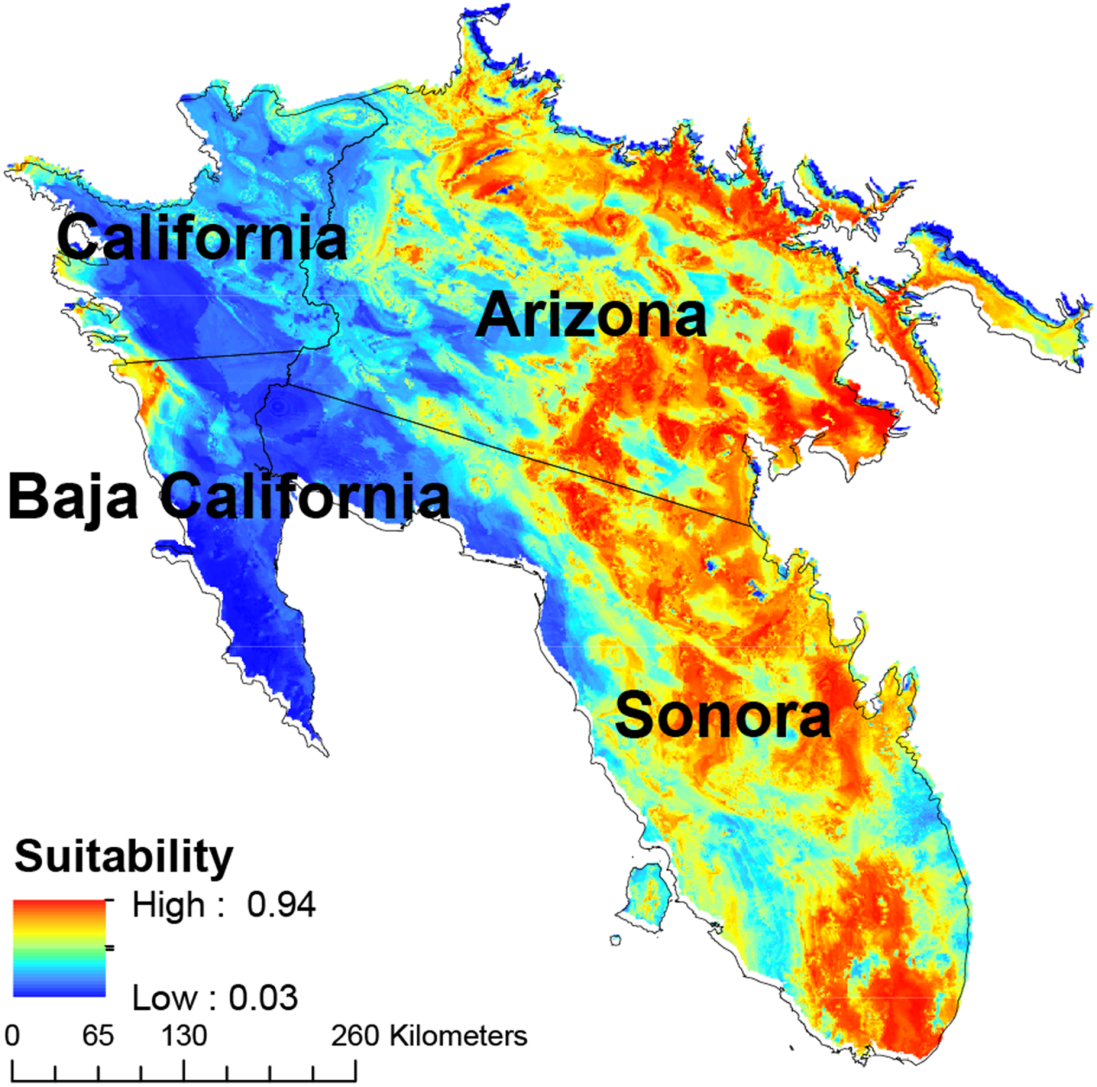
Map of habitat suitability of Saguaro. Geographical distribution of habitat suitability of Saguaro across the Sonoran Desert.

The eastern and southern areas of Arizona, USA, and Sonora, Mexico, showed the largest concentrations of areas with high suitability, with a secondary concentration in central Sonora state (Fig. 4). Cells with high habitat suitability were also concentrated in a northernmost part of the Sonora desert (Fig. 4).

**Figure 5:**
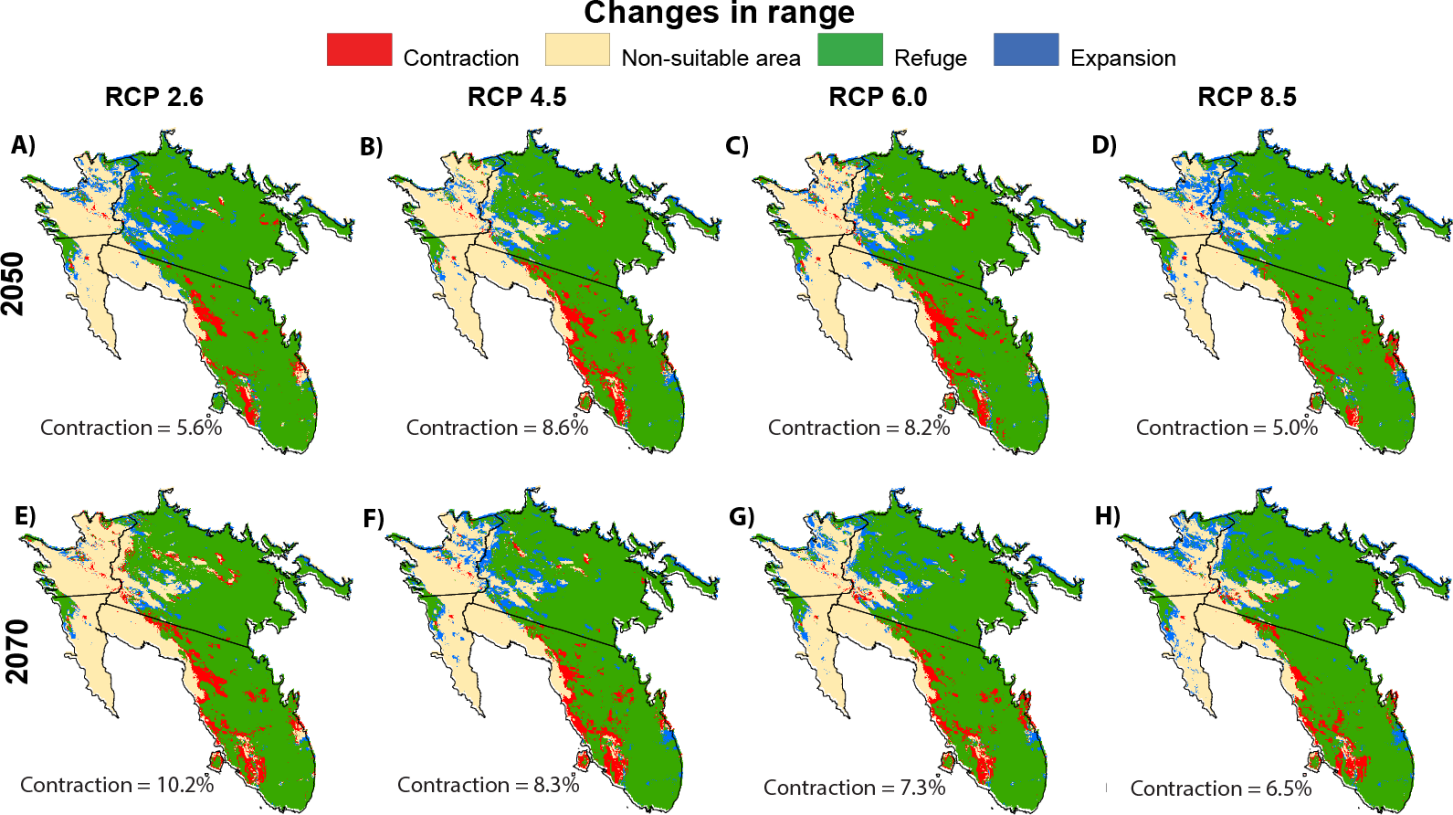
Potential changes. Geographical distribution of predicted actual Saguaro distribution (2017), according to the boosted tree regression model, and potential changes in ranges in saguaro’s range under future climate change scenarios for 2050 ((A–D) average for 2041–2060) and 2070 ((E–H) average for 2061–2080).

Model forecast under different RCPs indicated significant habitat suitability reductions across the species’ presence range and few opportunities for range expansion (Fig. 5). In all RCPs, models predicted a high contraction of suitable habitat. By 2050, RCPs predict a loss of 6.9%, on average, of the of currently suitable habitat, with values ranging from 5.6% (RCP 2.6) to 8.6% (RCP 4.5). Much of the contiguous loss of suitable habitat is on the western edge of saguaro’s range in Arizona, with a sizeable loss in a central patch of Arizona. This pattern continues into Mexico, with contractions on the western edge of Sonora, jetting into the mainland, and receding north from Sinaloa. The models projected little habitat suitability increases, most notably in Sonora State’s range. By 2070, this pattern of habitat suitability loss is projected to continue and worsen with an additional 1.2% contraction from 2050. The greatest expected change appears to be enlarging inland patches of unsuitable habitat from Arizona to Mexico. Our models suggest a moderate growth in habitat suitability in the northward expansion from saguaro’s actual range. We also observed an expansion in the center of the Sonora State.

**Figure 6:**
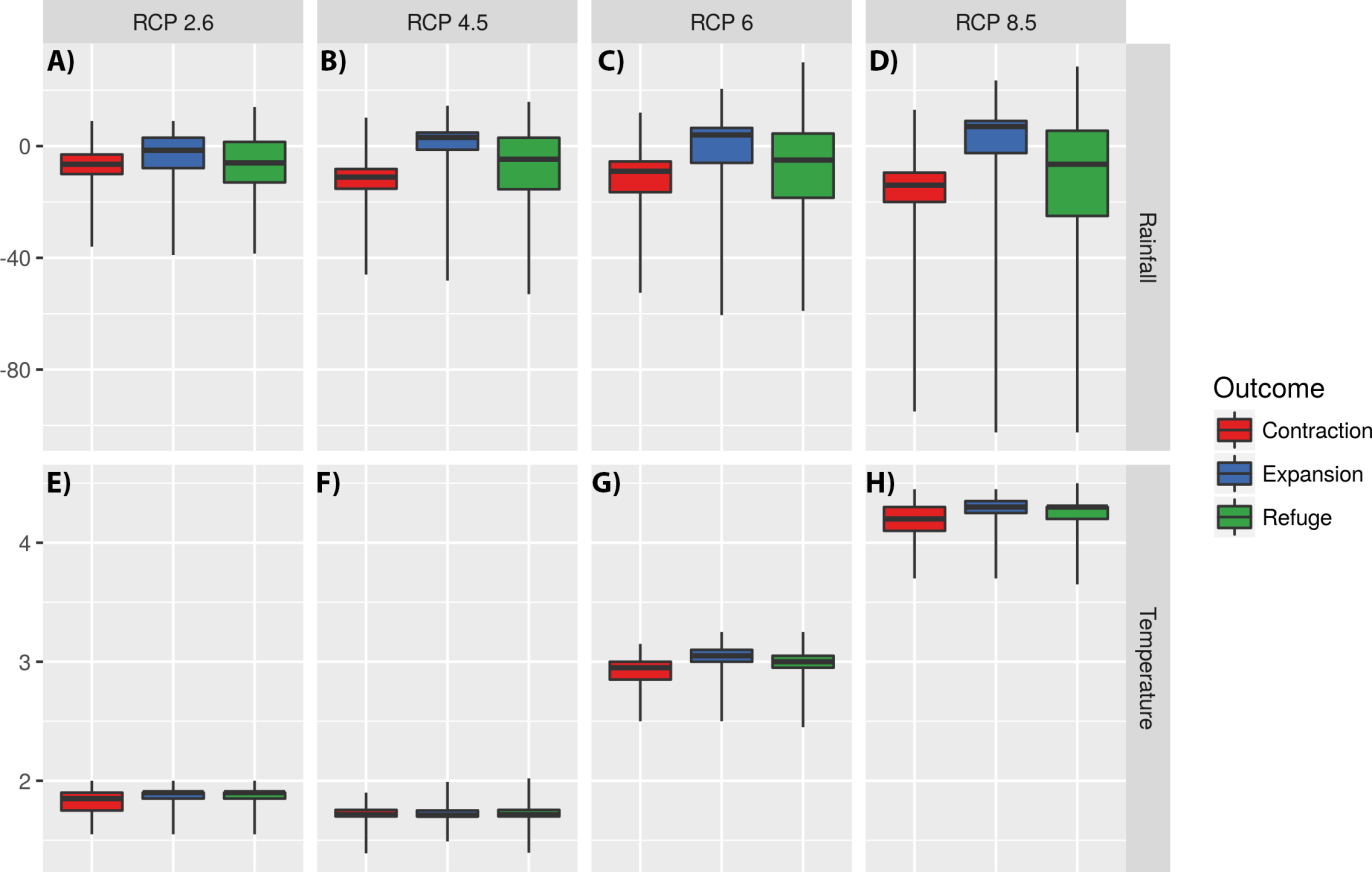
Impact of changes in annual meant temperature and annual precipitation on defining expansion, refuge, and contraction areas. The impact of changes in annual precipitation (A–D) and annual mean temperature (E–H) on defining expansion, refuge, and contraction areas for four Representative Concentration Pathways (RCPs): RCP2.6, RCP4.5, RCP6.0 and RCP8.5. *Y* values represent the differences in temperature and rainfall between today and 2070. The central line represents the median. The lower and upper hinges correspond to the first and third quartiles (the 25th and 75th percentiles). Upper and lower whiskers extend to the largest and lower value, but no further than 1.5 multiplied by the distance between the first and third quartiles. Outliers were not plotted for clarity.

Models also suggest that temperature has low impact on defining expansion and contraction areas for RCPs 2.5 and 4.6, where increased rainfall is the main factor explaining the increase of habitat suitability (Fig. 6). For RCPs 6.0 and 8.5 expansion areas seem to be mostly explained by an increment in both temperature and rainfall (Fig. 6).

## Discussion

For first time, we used BRT models to describe the relationships of environmental variables and the habitat suitability of the iconic saguaro across the Sonoran Desert, and predicted habitat suitability change under climate warming for four different RCPs.

In general, the predictive performance of boosted regression trees depends on the model parameters, such as *lr* and *tc*. BRT models required a large *tr* value to achieve a minimum predictive error. After testing different combinations of parameters, three models emerged as best candidates: they shared the same *tr* (4), *bf* (0.50) and *AUC* (0.87). We selected the model with smaller *lr* (0.005), because small values for *lr* results in a slower learning and requires a higher number of trees to improve the predictive error (De’ath, 2007). Also, small *lr* values shrinks the contribution of each tree and reliably estimate the response (Elith, Leathwick & Hastie, 2008). BRT models were also expected to be affected by target distance, as it happened. Models with smaller target distance, and therefore larger sample size, produced higher AUC values than models with larger target distances, which also produced lower explained deviance values.

We found that the habitat suitability of the saguaro is strongly related with climate variables, which also agrees with previous studies performed at local extent (Parker, 1993; Drezner & Balling, 2002). According to BRT’s variable importance measures, annual precipitation had the strongest influence on the habitat suitability of the saguaro. Overall, the probability of occurrence increased with the annual precipitation, rising steeply and uniformly up to 300 mm, followed by a steep decrease and a stationary phase. Several other studies have identified precipitation as a key factor for the demography of saguaros. Turner, Bowers & Burgess (1995) observed that the saguaro grows in areas of the Sonoran Desert where summer rainfall is substantial. Drezner (2006) reported that the reproductive success of the saguaro is closely related with global and regional-scale variations and increases in rainfall. Precipitation is also related with patterns in saguaro establishment and survival (Winkler et al., 2018). The lack of water is pointed as a major factor affecting cacti mortality, probably because water limitation can reduce the survivorship of young and juvenile individuals (Pierson & Turner, 1998). Furthermore, desert ecosystems of western United States and Northern Mexico are particularly susceptible to climate variability and specifically to drought (Archer & Predick, 2008).

According to our model, the max temperature of warmest month had a strong influence on the habitat suitability for the saguaro. Overall, habitat suitability dramatically increased when the maximum temperature of the warmest month went beyond 36 °C. This relationship may occur because of the saguaro is well adapted to the harsh temperature conditions of the Sonoran Desert (Franco & Nobel, 1989). Temperature has been identified as one of the most important factors for the regeneration and population viability of the saguaro (Turner, Bowers & Burgess, 1995; Drezner, 2006), since it plays a key role in driving the establishment and survival of young saguaros, maintaining its distribution over time (Turner et al., 1966; Nobel, 1982). On the other hand, saguaros are sensitive to extended periods of subfreezing temperatures (Nobel, 1982), and catastrophic freeze events have been reported to increase the mortality of the saguaro (Orum, Ferguson & Mihail, 2016).

Our models show potential impacts of climate change on saguaro’s habitat suitability in the Sonoran Desert, a result that is consistent with previous analyses of climate change in desert ecosystems (SDNIMP, 2010; Munson et al., 2012). All models projected onto different RCPs predict a reduction in habitat suitability for the saguaro. Specifically, results indicate that the eastern and central parts of Mexico, and especially the Sonora State, are more sensitive to changes, and face large habitat suitability decreases.

The impacts of climate change on the distribution of the saguaro have recently been reported at the Saguaro National Park, Arizona (Swann et al., 2018). Climate change seems to affect the saguaro directly through increased drought, the occurrence of extended, extreme freezing events, and indirectly because warmer winters temperatures may enhance the spread of exotic species, such as buffelgrass (*Cenchrus ciliaris*; Swann et al., 2018). Also, drought directly promotes the decline of saguaro density and growth, and reduces perennial shrub and tree cover (nurse plants), which help to protect saguaro from extreme temperatures (Archer & Predick, 2008). We add on to previous studies, showing for the first time the potential changes in the habitat suitability of *C. gigantea*, under future climate change scenarios, for the whole Sonoran Desert area. Although much work remains to be done to evaluate the effect of climate change on the distribution of saguaro across the Sonoran Desert, our findings provide a strong reason to engage in that work. Because the saguaro distribution is so poorly documented, conservation planners need reliable assessments to monitor the reduction in suitability at Sonoran Desert.

## Conclusion

In this study, we used boosted regression trees to investigate effects of climate change on the saguaro habitat suitability, and to explore the complex relationship between environmental factors and saguaro distribution in the Sonora Desert ecosystem at a regional extent. Based on our results, we reached three conclusions: (1) the performance of BRT algorithms varied with the selection of BRT parameters. Overall, BRT models performed well, which reinforces its use for typical ecological analyses (Elith, Leathwick & Hastie, 2008). As indicated by cross-validation analysis, BRT is a useful algorithm for analyzing and predicting ecological data (De’ath, 2007). (2) BRT models identified precipitation and temperature as the main drivers of the habitat suitability for the saguaro in the Sonoran Desert. (3) Although previous studies have reported impacts of climate change on the saguaro, this study is the first attempt to identify potential impacts of climate change on the saguaro’s habitat suitability across its whole range. Previous studies on the possible effects of climate change on saguaro distribution have mostly focused on local scales (Turner et al., 1966; Pierson & Turner, 1998; Archer & Predick, 2008; Swann et al., 2018), while this study is focused on a regional scale. Regardless of the RCP used, models predict a decrease in the saguaro’s habitat suitability across the study area. Also, our results allow us to conclude that under warming conditions an increase in precipitation is required to ensure a high habitat suitability for saguaros. Because saguaros are much more resistant to extended drought than many other species (Swann et al., 2018), we suggest that elucidating the patterns and drivers of species distribution change under climate warming can provide key ecological knowledge necessary to conserve species at the Sonoran Desert.

##  Supplemental Information

10.7717/peerj.5623/supp-1 Figure S1 Cluster analysis of environmental variables

10.7717/peerj.5623/supp-2 Table S1 List of candidate variables used in the cluster analysis

10.7717/peerj.5623/supp-3 Data S1 Raw data Occurrences records on the saguaro distribution and pseudo-absences used to calibrate and fit species distribution models.

10.7717/peerj.5623/supp-4 Appendix S1 Cluster analysis Cluster analysis including 26 environmental variables as potential predictors of saguaro habitat suitability. Variables are: Bio1, annual mean temperature; Bio2, mean diurnal range; Bio3, Isothermality; Bio4, temperature seasonality; Bio5, max temperature of warmest month; Bio6, min temperature of coldest month, Bio7; temperature annual range; Bio8, mean temperature of wettest quarter; Bio9, mean temperature of driest quarter; Bio10, mean temperature of warmest quarter; Bio11, mean temperature of coldest quarter; Bio12, annual precipitation; Bio13, precipitation of wettest month; Bio14, precipitation of driest month; Bio15, precipitation seasonality; Bio16, precipitation of wettest quarter; Bio17, precipitation of priest quarter; Bio18, precipitation of warmest quarter; Bio19, precipitation of coldest quarter; Pradm, Mean potential solar radiation, Pradsd, standard deviation of the potential solar radiation, Topo, topographic openness index and Topw, topographic wetness index.

## Additional Information and Declarations

### Data Availability

The following information was supplied regarding data availability: The raw data is available in the Supplemental File.
