# Supplementary figures and images for: Potential changes in the distribution of Carnegiea gigantea under future scenarios
Source: PeerJ. 2018 Sep 19;6:e5623. doi: 10.7717/peerj.5623 (PMC6151114; doi:10.7717/peerj.5623)

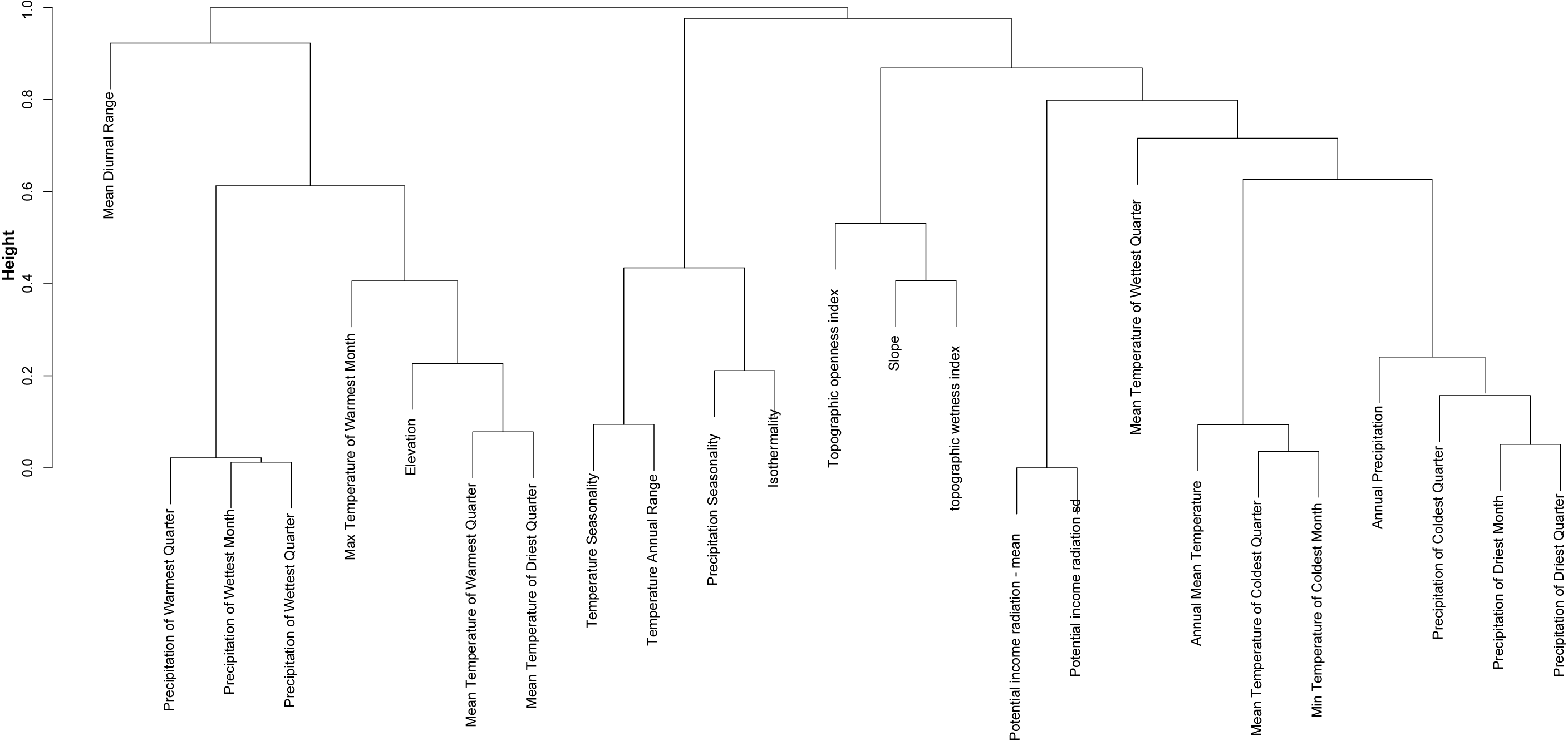

Supplement: Figure S1 [file peerj-06-5623-s001.png]

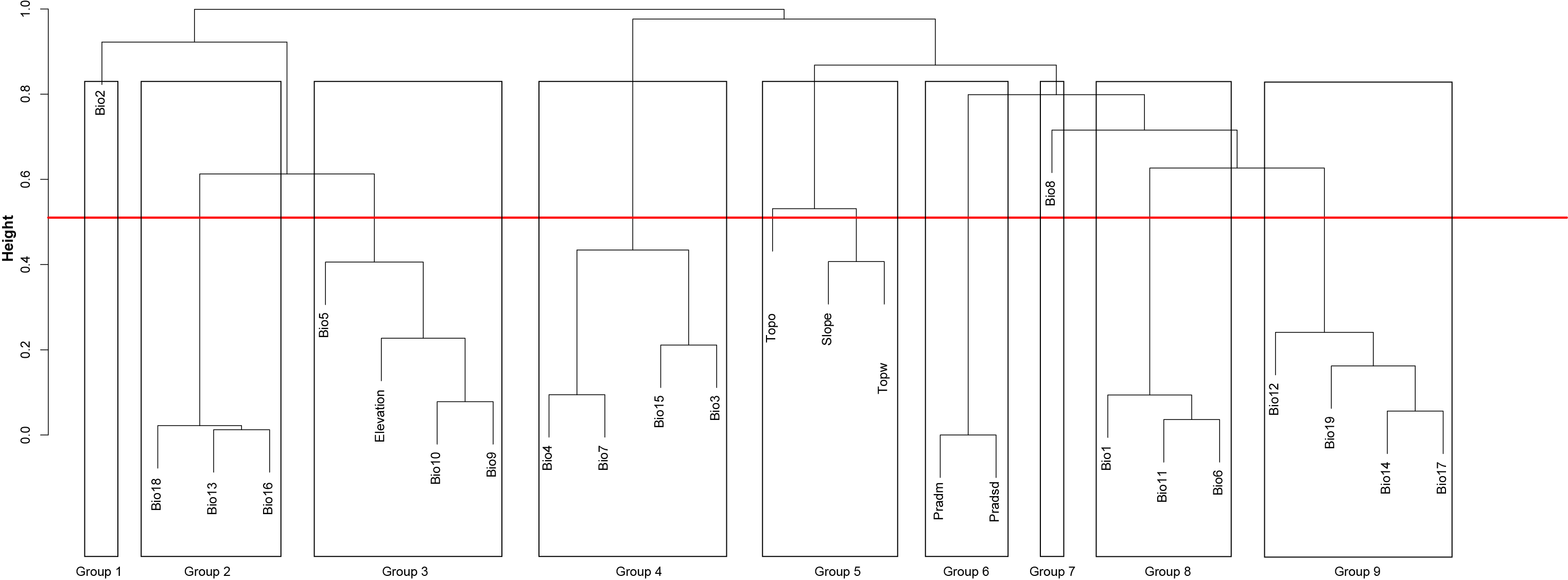

Supplement: Appendix S1 — Cluster analysis including 26 environmental variables as potential predictors of saguaro habitat suitability. Variables are: Bio1, annual mean temperature; Bio2, mean diurnal range; Bio3, Isothermality; Bio4, temperature seasonality; Bio5, max temperature of warmest month; Bio6, min temperature of coldest month, Bio7; temperature annual range; Bio8, mean temperature of wettest quarter; Bio9, mean temperature of driest quarter; Bio10, mean temperature of warmest quarter; Bio11, mean temperature of coldest quarter; Bio12, annual precipitation; Bio13, precipitation of wettest month; Bio14, precipitation of driest month; Bio15, precipitation seasonality; Bio16, precipitation of wettest quarter; Bio17, precipitation of priest quarter; Bio18, precipitation of warmest quarter; Bio19, precipitation of coldest quarter; Pradm, Mean potential solar radiation, Pradsd, standard deviation of the potential solar radiation, Topo, topographic openness index and Topw, topographic wetness index. [file peerj-06-5623-s004.png]
