# Supplementary material for: Potential changes in the distribution of Carnegiea gigantea under future scenarios
Source: PeerJ. 2018 Sep 19;6:e5623. doi: 10.7717/peerj.5623 (PMC6151114; doi:10.7717/peerj.5623)
Supplement: Table S1 [file peerj-06-5623-s002.docx]

| **Main factors** | **Code** | **Description** | **Source** |
| --- | --- | --- | --- |
| **Climate** | Bio1 | Annual Mean Temperature | Hijmans et al. 2005 |
|  | Bio2 | Mean Diurnal Range (Mean of monthly (max temp - min temp)) |  |
|  | Bio3 | Isothermality (BIO2/BIO7) (* 100) |  |
|  | Bio4 | Temperature Seasonality (standard deviation *100) |  |
|  | Bio5 | Max Temperature of Warmest Month |  |
|  | Bio6 | Min Temperature of Coldest Month |  |
|  | Bio7 | Temperature Annual Range (BIO5-BIO6) |  |
|  | Bio8 | Mean Temperature of Wettest Quarter |  |
|  | Bio9 | Mean Temperature of Driest Quarter |  |
|  | Bio10 | Mean Temperature of Warmest Quarter |  |
|  | Bio11 | Mean Temperature of Coldest Quarter |  |
|  | Bio12 | Annual Precipitation |  |
|  | Bio13 | Precipitation of Wettest Month |  |
|  | Bio14 | Precipitation of Driest Month |  |
|  | Bio15 | Precipitation Seasonality (Coefficient of Variation) |  |
|  | Bio16 | Precipitation of Wettest Quarter |  |
|  | Bio17 | Precipitation of Driest Quarter |  |
|  | Bio18 | Precipitation of Warmest Quarter |  |
|  | Bio19 | Precipitation of Coldest Quarter |  |
| **Topography** | Elevation | Range in elevation | ISRIC, 2013; Hengl et al., 2014; WorldGrids (2018) |
|  | Slope | Slope |  |
|  | Topw | topographic wetness index |  |
|  | Topi | topographic openness index |  |
|  | Rad_mean | Mean potential incoming radiation |  |
|  | Rad_sd | Potential incoming radiation - Standard deviation |  |
